# Depression, Anxiety, and Social Environmental Adversity as Potential Modulators of the Immune Tumor Microenvironment in Breast Cancer Patients

**DOI:** 10.3390/medsci9020046

**Published:** 2021-06-21

**Authors:** Eida M. Castro-Figueroa, Karina I. Acevedo, Cristina I. Peña-Vargas, Normarie Torres-Blasco, Idhaliz Flores, Claudia B. Colón-Echevarria, Lizette Maldonado, Zindie Rodríguez, Alexandra N. Aquino-Acevedo, Heather Jim, María I. Lazaro, Guillermo N. Armaiz-Peña

**Affiliations:** 1 Clinical Psychology Program, School of Behavioral and Brain Sciences, Ponce Health Sciences University, Ponce, PR 00716, USA; kacevedo15@stu.psm.edu (K.I.A.); cpena@psm.edu (C.I.P.-V.); normarietorres@psm.edu (N.T.-B.); mlazaro17@stu.psm.edu (M.I.L.); 2 Division of Mental Health, Ponce Research Institute, Ponce, PR 00716, USA; 3 Division of Women’s Health, Ponce Research Institute, Ponce, PR 00716, USA; iflores@psm.edu (I.F.); garmaiz@psm.edu (G.N.A.-P.); 4 Division of Microbiology, Department of Basic Sciences, School of Medicine, Ponce Health Sciences University, Ponce, PR 00716, USA; 5 Division of Pharmacology, Department of Basic Sciences, School of Medicine, Ponce Health Sciences University, Ponce, PR 00716, USA; ccolon13@stu.psm.edu (C.B.C.-E.); aaquino18@stu.psm.edu (A.N.A.-A.); 6 Division of Cancer Biology, Ponce Research Institute, Ponce, PR 00716, USA; lizettemaldonado@gmail.com (L.M.); zrodriguez@psm.edu (Z.R.); 7 Department of Health Outcomes and Behavior, H. Lee Moffitt Cancer Center and Research Institute, Tampa, FL 33612, USA; Heather.Jim@moffitt.org

**Keywords:** tumor microenvironment, breast cancer, depression, anxiety, immune cells, social environmental adversity

## Abstract

*Background*: Mounting data suggest that exposure to chronic stress is associated with worse breast cancer outcomes. This study aimed to explore the impact of social environmental adversity (SEA, e.g., child abuse, crime, sexual, and physical violence), depressive symptomatology, and anxiety on immune cell infiltration into the breast tumor microenvironment. *Methods*: Participants (*n* = 33) completed a series of surveys assessing depression and anxiety symptoms, adverse childhood events (ACE), and trauma history. Tumor-associated macrophages (CD68+), B cells (CD19+), and T cells (CD3+) were identified by immunohistochemical analyses of formalin-fixed paraffin-embedded tumor samples and quantified. Spearman rank tests were used to explore the relationships between the variables studied. *Results:* Exposure to SEA was high (ACE = 72%, exposure to crime = 47%, and exposure to physical/sexual assault = 73%) among participants. Moreover, 30% reported a comorbid history of depression and ACE; 39% reported one or more traumatic events, and clinically significant depression symptomatology, while 21% reported trauma history and significant anxiety symptomatology. Increased tumor-infiltrating B cells were significantly correlated with exposure to crime, anxiety symptoms, and exposure to an ACE. The ACE plus anxiety group presented the highest infiltration of B cells, T cells, and macrophages. *Conclusion*: These findings support a role for SEA, anxiety symptoms, and depression as potential modulators of the immune tumor microenvironment in breast cancer.

## 1. Introduction

Breast cancer is the most common cancer diagnosed in women, with an estimated 2.3 million new cases each year globally [1]. Worldwide, breast cancer is the fifth leading cause of cancer-related mortality and the leading cause of cancer-related deaths in most Latin American countries [1] including Puerto Rico (a USA territory with a large Hispanic population) [1,2]. Even though several known global health disparities drive systemic breast cancer inequities (e.g., access to care), little is known about the role of exposure to social environmental adversity in breast cancer on clinical outcomes. Of note, social environmental adversity (SEA) is a term used to group the exposure of lifetime traumatic experiences such as child abuse, physical and sexual abuse, natural disasters, and domestic violence, among other traumatic experiences that lead to altered behavioral states, such as chronic stress [3].

Mounting preclinical and clinical evidence supports a key role for the sustained activation of adrenergic-mediated signaling as a driver of tumor growth and progression, as well as decreased survival in various malignancies, including breast cancer [4,5,6,7,8]. Moreover, according to the literature, the global prevalence of depression hovers around 30% among breast cancer patients [9]. Chronic sympathetic nervous system (SNS) activation induces inflammatory processes in the tumor microenvironment (TM) of several cancers [10]. These (SNS and TM) are associated with the induction of growth-promoting signals primarily mediated by b-adrenergic receptors in tumor cells [11]. Thus, the SNS is a critical pathway by which chronic psychological stress can promote tumor growth [4].

Studies have identified possible environmental and lifestyle factors contributing to breast cancer diagnosis or disease progression, such as dietary choices, physical inactivity, and alcohol consumption [12,13,14]. There is growing evidence suggesting that depression and anxiety symptomatology in cancer patients are related to tumor and treatment-related inflammatory processes [12,13,14] that are known to induce tumor growth [15,16]. This is a crucial area to study due to the potential relationship between psychological distress and cancer progression.

Emerging evidence suggests that Hispanics are at higher risk of being exposed to traumatic SEA-related events than Non-Hispanic Whites (NHWs) in the form of child maltreatment and witnessing domestic violence [17]. Several studies have demonstrated a link between depression, anxiety [18], SEA, and markers of systemic inflammation [3] consistent with the Social Signal Transduction Theory of Depression [3]. This theory postulates that exposure to SEA modulates the systemic inflammatory activity and, in turn, induces symptoms of anxiety and depression. The social stressors most relevant to anxiety and depression, which are also linked to inflammation, are adverse childhood events (ACE), interpersonal loss, and social isolation [3]. Studies have shed light on the possibility that this link could lead to worse cancer health outcomes [19,20,21,22,23]. For example, chronic stress in breast cancer patients was related to accelerated disease progression [6,24,25].

Various studies linked chronic stress, exposure to stressful events, and inflammation markers among breast cancer (BC) patients [26,27,28,29,30]. For example, a longitudinal pilot study found that participants with BC (n = 20) exposed to childhood trauma had significant depressive symptomatology and fatigue levels. These participants exhibited increased NF-kB pathway activation in peripheral blood mononuclear cells and had higher baseline levels of C-reactive protein, IL-6, and IL-1 in plasma [26]. Another study determined that BC patients (n = 16) with high stress (as measured by the Impact of Event Scale) after surgery exhibited increased myeloid-derived suppressor cells (MDSC) in the blood [27]. 

These cells help assemble the tumor microenvironment through various mechanisms (e.g., chemokine-regulated recruitment, the overproduction of cytokines, and the induction of T_reg_ cells) and contribute to tumor antigen-specific T cell non-responsiveness [28]. A longitudinal study revealed that a lack of social support (a form of social isolation) predicted higher IL-6 levels and depressive symptoms among BC survivors [29]. Furthermore, early relational stress exposure (represented by non-optimal parental bonding, such as rejection and neglect) was significantly associated with a positive lymph node status in a group of BC patients scheduled for surgery [30].

In sum, there is growing evidence supporting systemic inflammation as a common factor in depression, chronic psychosocial stress, and the sustained activation of the sympathetic nervous system. Recent work has highlighted the role of stress hormones in tumor progression [31]. However, very little is known about the interplay between SEA, depression, and anxiety on immune cells infiltration into the breast tumor microenvironment [32,33]. To address this gap, our study aimed to investigate the relationship between comorbid depression, anxiety, SEA, and immune cell infiltration (macrophages, B cells, and T cells) in the tumor microenvironment of Hispanic breast cancer patients.

## 2. Methods

Eligible participants were: (1) 21 years of age and older (the adult legal age in Puerto Rico), (2) females diagnosed with breast cancer, (3) donated tumor tissue for research, (4) not diagnosed with metastatic disease, and (5) had no documented or observed cognitive, visual, or physical impairment that would interfere with study participation. A total of 33 participants (power > 0.80, r > 0.35, a = 0.5, two-sided) were recruited through the Ponce Research Institute–Puerto Rico Biobank (PRBB). The PRBB is a biorepository focused on biobanking biological specimens (fresh frozen tissues, paraffin blocks, high-quality tumor-derived RNA, and genomic DNA) of Hispanic cancer patients from Puerto Rico. The PRBB collects clinical and epidemiological data using medical chart reviews and self-reported surveys from patients recruited through a network of collaborators, including hospitals and oncology clinics. Eligible participants received an appointment to complete the informed consent (which included the PRBB permission to provide a specimen) and the data collection process.

After signing the informed consent, participants completed a series of self-reported questionnaires that included: (1) sociodemographic, lifestyle, and clinical information, (2) the Patient Health Questionnaire-9 (PHQ-9) [34], (3) the General Anxiety and Depression-7 Scale (GAD-7) [35], (4) the Trauma History Questionnaire–Spanish Version (THQ) that measures exposure to three domains of traumatic events, such as sexual/physical abuse, exposure to crime, general disaster, and trauma [36], and; (5) the Adverse Childhood Experience Questionnaire (ACEQ) that measures exposure to the ten most common childhood adverse events [37]. 

The behavioral data collection process took approximately 60 min; participants were provided a 5-min break every 30 min or upon request. After completion, participants were provided with a stipend to compensate for their time and effort. The PRBB provided breast tumor samples (formalin-fixed paraffin-embedded) and clinical data extracted from the patients’ medical charts, such as breast tumor stage, tumor size, and lymph node status.

Formalin-fixed paraffin-embedded tumor sections were stained for macrophage infiltration using CD68 (1:200; Dako, M0814), B cell infiltration with CD19 (1:100; Abcam, ab134114), and pan T cell infiltration with CD3 (1:100; Abcam, ab5690). Briefly, sections were fixed in 4 °C acetone for 10 min and rinsed with 1× PBS. Endogenous peroxide activity was blocked using 0.3% H202 for 10 min, followed by antigen retrieval with Citrate-EDTA buffer pH 6.2 (CD68, CD3) or Tris-EDTA buffer pH 9.0 (CD19) for 40 min in a preheated water bath (95–99 °C). The slides were incubated with protein block solution for 1 h at room temperature. 

Tissues were incubated with the respective primary antibodies overnight at 4 °C in a humidity chamber. Protein block, secondary antibody, and 3,3′ Diaminobenzidine (DAB) in the Super Sensitive Link Label IHC kit (LP000-ULE, BioGenex, Fremont, CA) were used according to the manufacturer’s instructions. All primary and secondary antibodies were diluted in PBS. Counterstain was performed using hematoxylin (Sigma, GHS316, St. Louis, MO, USA) for 5 s, followed by washes with running tap water for 5 min. The slides were dried, mounted, and blindly scored by investigators. For each patient sample, five random 20× high power fields were manually quantified for each marker with ImageJ software and averaged per slide.

Univariate descriptive analyses were used to explore the comorbid rates of depression (PHQ-9) scores ≥ 5 and self-reported depression diagnosis in the past) and exposure to SEA (defined as having at least one ACE or one “yes’ item reported in the THQ). The same analyses were employed to estimate the comorbid rates of anxiety (GAD-7 scores ≥ 5) and exposure to SEA (ACE ≥ one + THQ ≥ one).

We analyzed these data using Spearman’s rank correlation coefficient to determine an association between immune cell infiltration and comorbid depression, anxiety, or SEA. We compared continuous variables with the Mann–Whitney test. Categorical variables were assessed using the chi-square test. For this study, sample calculation was performed using the G*Power tool. For each statistical assessment power > 0.80 to detect r > 0.35 with n = 32, a = 0.5, and a two-sided test. A *p*-value of 0.05 was considered statistically significant.

## 3. Results

Table 1 summarizes the participants’ sociodemographic and clinical characteristics and correlations among behavioral outcome variables (depression, anxiety, and exposure to SEA). The participants’ mean age was 62.8 (SD = 10.58). The majority of women reported being married (60.6%) and retired from employment (45.5%), followed by unemployed (18.2%) and currently employed (18.2%). Most participants reported an annual income ranging from $19,001–$35,000 (45.5%), followed by an income of equal or less than $19,000 (42.4%). 

Moreover, 56.6% of the participants reported that their annual income was not enough to cover their basic family needs. Although Puerto Rico is considered a wealthy region of Latin America, as a USA territory, it has a significantly higher poverty rate (43.5%) compared to other USA territories (US Virgin Islands = 22.4% and Guam = 22.9%) [38]. Regarding clinical characteristics: 21.2% of participants were diagnosed with a T2 cancer stage, followed by T1c (15.2%). Lastly, 21.2% of the sample reported positive lymph node status.

Regarding depressive symptomatology as assessed by PHQ-9, 20% reported depressive symptoms (from mild to severe), and 21% reported experiencing anxiety symptoms (GAD-7). The THQ results showed that 100% of participants reported at least one SEA event, and 72.7% reported exposure to two or more SEA events. Additionally, 47% reported exposure to crime events, 94% reported exposure to general disaster/trauma events, and 73% reported exposure to physical/sexual abuse events (33% was sexual abuse and 67% was physical abuse). A further 30% reported a comorbid history of depression symptoms and ACE scores, 39% reported comorbid trauma history (THQ) and depression symptoms, while 21% reported a history of trauma and anxiety symptoms. Meanwhile, 72% reported exposure to one or more ACE (ACEQ), with 27% reporting four or more events for a mean score of 2.36 (SD = 2.56).

Data (Table 1) also showed that depression symptoms plus ACE scores (*p* < 0.001) and a comorbid history of depression plus THQ scores (*p* < 0.01) significantly correlated with reporting a mental health diagnosis. Sixty-one percent of participants who reported having received mental health services in the past also reported comorbid THQ and depression. In addition, past mental health services were significantly correlated to comorbid depression symptoms plus ACE scores (*p* = 0.01), to comorbid depression symptoms plus THQ scores (*p* < 0.01), and to a history of depression alone (*p* < 0.01).

On the other hand, we found a positive, statistically significant relationship between alcohol consumption in the past six months and comorbid trauma history and history of depression diagnosis (*p* = 0.01). A total of 70% of participants reporting alcohol consumption also reported comorbid trauma history and depression diagnosis.

**Figure 1:**
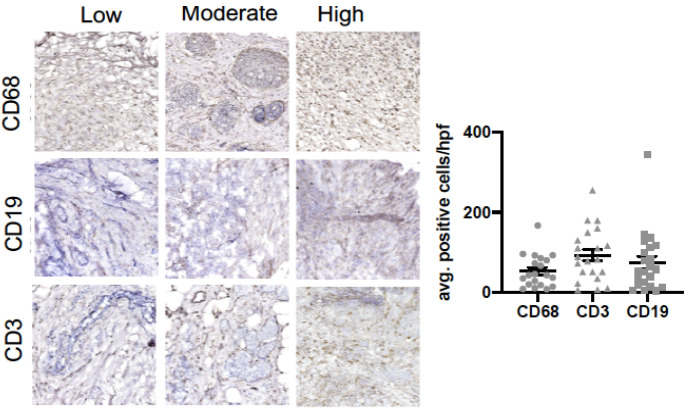
*B cell, T cell and macrophage infiltration in breast tumor samples*. Immunohistochemical analysis of breast tumor samples showing expression of CD68+ (macrophages), CD19+ (B cells) and CD3+ (T cells) cells.

We also assessed if a relationship existed between intra-tumoral infiltrates representative of the innate (macrophage) and adaptive (B cell and T cell) immune response and clinicopathological variables (Figure 1 and Table 2). Figure 1 shows representative images of macrophages (CD68), B cells (CD19), and T cells (CD3) in our cohort. Our data show that an increased tumoral infiltration of T cells and decreased B cell infiltration were positively associated with increased age (*p* < 0.01). Macrophage infiltration was associated with postmenopausal status (*p* < 0.05), active chemotherapy (*p* < 0.01), and positive lymph nodes (*p* < 0.05). Macrophage infiltration was associated with past mental health services (*p* < 0.05) and lack of social support (*p* < 0.05).

Aiming to explore the impact of SEA on the infiltration of immune cells into the tumor microenvironment, we explored potential correlations between SEA exposures, depression, and anxiety with macrophages, T cells, and B cells (see Table 3). In this context, we found that increased macrophage infiltration was significantly associated with depression symptoms (*p* < 0.05), while increased B cell infiltration was significantly associated with exposure to ACE (*p* < 0.05).

Table 3 illustrates the correlations between depression, anxiety, and categories of exposure to SEA. Our data show that increased B cell infiltration was significantly associated with comorbid exposure to crime and anxiety symptoms (*p* < 0.05). There was a statistically significant relationship between increased T cell infiltration and comorbid exposure to physical/sexual abuse and anxiety symptoms (*p* < 0.05).

**Figure 2:**
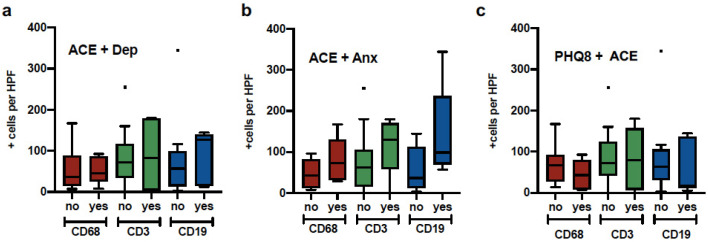
*Macrophage, T cell and B cell expression in patients with psychological distress*. Tumor samples from breast cancer patients were quantified for Macrophage (CD68), B cells (CD19) and T cells (CD3) and divided into groups a) Elevated ACE and history of depression, b) Elevated ACE and GAD7, c) Elevated PHQ8 and Elevated ACE.

Figure 2 illustrates the distribution of comparisons between macrophages (CD68+ cells), T cells (CD3+ cells), and B cells (CD19+ cells) with ACE plus depression, ACE plus anxiety, and history of trauma (THQ) plus ACE. Analyses did not yield significant results; however, the ACE plus anxiety group presented the most infiltration of the three types of immune cells (*p* = 0.06).

## 4. Discussion

The goal of this study was to examine whether breast cancer patients exposed to social environmental adversity (SEA, e.g., child abuse, crime, and sexual and physical violence) and reporting symptoms of depression and anxiety correlated with immune cells infiltration in the breast tumor microenvironment. Our work builds from existing data suggesting that the physiological stress response via chronic activation of the SNS and the hypothalamic–pituitary–adrenal (HPA) axis promotes inflammatory responses that lead to tumor growth and progression [6,23,24,39].

In our study, the rates of anxiety and depression symptoms among study participants were similar to those reported previously by breast cancer patients in a systematic review [40]. The rates of ACE were comparable to Hispanic women in the US [41,42]. However, our sample reported higher rates of ACE events (at least one event) when compared to ACE events reported by other groups of breast cancer patients [43,44]. Our sample reported an outstanding rate of four or more ACE. This outcome can be related to the increased risk of developing chronic conditions compared to Puerto Ricans without cancer included in other studies, such as the Study of Latino (SOL) cohort (27%, vs. 38.1%, respectively) [41]. 

Moreover, this risk is higher than for overall Hispanic men (25.8%) and other Hispanic sub-ethnicities (e.g., Cubans: 20.2% and South Americans: 24.0%) included in the SOL [45] the other hand, rates of exposure to SEA (100%, at least one SEA) were high. Interestingly, 73% reported lifetime exposure to physical/sexual abuse, and, out of these, 33% reported being exposed to sexual assault, and 67% reported being exposed to physical assaults. While the rates of lifetime sexual assault (33%) in our study were similar to the US national rates among Hispanic women (35.6%) [45]; the rate of physical assault in our sample was significantly higher than the rate of physical abuse reported by Hispanic women who participated in the Sexual Assault Among Latinas Study (SALAS study, 67% vs. 25.6%, respectively) [46]. 

Likewise, the rates of overall physical/sexual assaults were higher in our sample of Hispanic breast cancer patients when compared to Hispanic women in the SALAS study (73% vs. 66.2%, respectively) [47]. Overall, these findings suggest that, as a whole, our study sample was similar to the general and Hispanic population in terms of the rates of anxiety and depression symptoms, ACE, and sexual assault. However, higher rates of exposure to physical/sexual assault exposure among our sample of Hispanic breast cancer patients warrants further research to elucidate if these rates are comparable to the general population of women living in Puerto Rico.

We found a positive relationship between depression symptoms and the infiltration of macrophages into the tumor microenvironment. Macrophages (M1) are significant contributors to neuroinflammatory processes linked to major depression symptoms [48]. On the other hand, ACE exposure was significantly correlated with increased B cell infiltration. Compared to patients not reporting ACE and anxiety comorbidity, the ACE + Anxiety group had elevated B cell, T cell, and macrophage infiltration, although this was marginally significant (*p* = 0.06). Moreover, anxiety symptoms combined with exposure to crime or exposure to physical and sexual abuse were significantly associated with B cell and T cell infiltration, respectively.

These findings pose a potential clinical and translational value as evidence supports the role of immune infiltrates in promoting breast tumor progression [46,47]. For example, CD4+ FOXP3+, CD4+, Th2 cells, macrophages, and MDSC cells promote growth by activating synergistic pathways between the immune microenvironment and tumor cells [49]. An analysis of almost 11,000 tumor samples showed that T regulatory cells and M0 and M2 macrophages were associated with a lack of response to chemotherapy, even after controlling for the estrogen receptor (ER) status [50]. Existing theories of tumor-infiltrating immune cells promoting tumor proliferation have identified other mechanisms of breast cancer invasion and metastasis [51]. 

For example, macrophages can induce cell migration and invasion through the epidermal growth factor (EGF) and promote angiogenesis by releasing VEGF. Another study suggested that immune cell-mediation through IL-4-expressing CD4+ T lymphocytes indirectly promotes breast cancer tumor invasion and further metastasis [48]. Furthermore, there is evidence that aberrant lymphocyte infiltration induced focal capsule disruptions to contribute to breast cancer tumor progression and invasion [48]. Recent studies have shown that B cells and regulatory B cells selectively promote breast cancer lymph node metastasis [52], implicating them in the metastatic process [53].

The primary limitation of this study is the small sample size that limits our ability to include potential confounders in multivariate analyses. Despite this limitation, sufficient power was apparent to detect significant correlations between the independent variable (exposure to SEA: ACE + THQ) and the primary outcomes (depression, anxiety, and immune cell infiltrates in the breast tumor microenvironment). Despite being a small sampled-sized study, we found that the rates of potential socio-behavioral risk factors (depression, anxiety, and SEA exposure) were similar to US general and Hispanic cancer patients and women populations. 

Future studies with larger patient numbers and a longitudinal design are needed to determine whether the combination of exposure to SEA, symptoms of anxiety, and depression can modulate the breast tumoral infiltration of cells from the innate and adaptive immune response. Another limitation of our study is the lack of additional strategies (RNA- or Exome-seq) or specific markers for determining immune cell sub-types (such as M1 or M2 macrophages, CD4+ or CD8+ T cells, among others). For example, we plan on performing RNA-seq on tumor samples to classify immune cell subtypes in tumor samples, as these analyses will complement and expand our current IHC-based approach. Moreover, follow-ups of larger-sampled work from our group will aim to determine if there are associations between ACE, anxiety, and depression with specific immune cell types, such as CD4+ or CD8+ T cells, M1/M2 macrophages, and specific B cells subtypes.

Despite these limitations, this study’s findings significantly contribute to the body of evidence regarding the link between depression, anxiety, SEA exposure, and breast cancer progression. Others have shown that chronic sympathetic nervous system activation leads to increased immune inflammation levels in tumors, including the breast cancer tumor microenvironment [9]. This process has been associated with the induction of growth-promoting signals primarily mediated by b-adrenergic receptors in tumor cells [11]. This is why a longitudinal study with a larger sample size is needed to elucidate the potential moderating role of exposure to SEA in the relationship between anxiety and depression symptoms and immune markers of breast cancer progression.

This study’s aims were partially guided by the Social Signal Transduction Theory of Depression [3], which was modified and applied to breast cancer patients. According to this theory, the innate immune response to physical predatory threats can be activated by modern-day social, symbolic, anticipated, and imagined threats and responses to contemporary chronic social-environmental adversity (e.g., ACE and physical and sexual abuse) that results in systemic inflammation [3]. A key component of this theory is the neuroinflammatory-sensitization to the adversity paradigm [3]. Neural responses to contemporary chronic SEA may up-regulate systemic inflammation [3]. 

Chronic SEA is represented in specific brain regions that initiate and modulate inflammatory activity and, in turn, induce cognitive, emotional, and behavioral symptoms of anxiety and depression [3]. As such, neural systems involved in processing social threats (e.g., the anterior insula and dorsal anterior cingulate cortex) have an anatomical connection to lower levels of the brain region, such as the hypothalamus and brainstem, which, in turn, influence systemic inflammation through activities of the HPA axis and the SNS [3]. Exposure to ACE has been associated with chronic activation of the HPA axis during sensitive developmental periods leading to epigenetic changes in the glucocorticoid receptor [52]. 

A life-course prospective cohort study (n = 1037) [54] found that the cumulative exposure to early life trauma was associated with elevated levels of C-reactive Protein (RR = 1.59; 95% CI = 1.02–2.50). Those who were exposed to ACE were more likely to experience more stress during adulthood [54]. A cancer diagnosis is a life-threatening event that can cause a tremendous emotional burden on the patient [55]. The theory’s neuroinflammatory-sensitization to adversity paradigm applied to the cancer experience may provide insights into known and novel biopsychosocial pathways (e.g., SEA, anxiety, depression, and adrenergic-mediated signaling) by which current and past exposure to chronic stress among breast cancer patients may lead to inflammatory phenotypes and cancer progression.

## 5. Conclusions

These findings support a role for SEA, symptoms of anxiety, and depression as potential modulators of the immune tumor microenvironment in breast cancer. These results provide the rationale for further studies in this area and represent a potential venue for intervention at the clinic. For example, our data supports developing further lager-sampled clinical studies to determine if exposure to SEA represent a risk factor for breast cancer progression. Our findings also support the clinical value of screening for (1) SEA, anxiety, and depression; and (2) inflammatory immune infiltrates in tumors, as these have been associated with worse outcomes in several cancers. 

Future studies may consider pharmacological and psychological approaches to prevent the effects of SNS or HPA activation on the immune tumor microenvironment among breast cancer patients exposed to SEA and presenting anxiety and depression symptoms. Future studies should also consider the implication of these findings among other racial/ethnic groups, especially among African American women with breast cancer, as evidence shows that this racial/ethnic minority presents high rates of exposure to SEA [14].

## Figures and Tables

**Table 1:** Participants’ characteristics and correlations with behavioral outcome variables.

Variables	Mean/SD, N/%	PHQ-8 (N = 33)	GAD-7 (N = 33)	HX. Depression (N = 33)	ACE (N = 33)	THQ (N = 33)	ACE + Depression (N = 33)	THQ + Depression (N = 33)
*Socio-Demographic Characteristics*								
Age	62.8 (SD = 10.58)	−0.223	−0.194		0.073	0.099		
Civil Status: Married	18 (57.6%)	1.312	0.788	0.001	0.418		0.034	0.137
With Employment	8 (24.2%)	0.792	1.676	0.090	1.161		1.676	0.015
Income (≤19,000)	14 (42.4%)	0.248	0.698	1.146	2.068		4.467 *	1.146
Income Enough (Yes)	14 (42.4%)	0.104	0.001	0.001	0.020		0.034	0.137
*Clinical Characteristics*								
Tumor Size (cm)	3.19 (SD = 2.8)	−0.242	−0.350		−0.096	−0.231		
Lymph Nodes	7 (21.2%)	0.046	0.377	0.326	0.326		0.029	0.326
Chemo-Therapy	9 (27.3%)	0.280	0.009	1.296	3.764*		0.836	0.836
Radiotherapy	3 (9.1%)	0.172549	0.014	0.647	0.416		0.027	0.416
Cancer Hormonal Therapy	6 (18.2%)	2.363	0.141	0.933	0.117		0.117	0.933
Menopause Status (Yes)	21(63.6%)	2.444	0.932	5.917 *	2.437		6.559 *	5.917 *
BMI	30.59 (SD = 4.8)	0.128	0.105		−0.102	0.055		
Mental Health Diagnosis (Yes)	9 (27.3%)	0.027	1.088	7.637 **	4.641 *		13.206 **	7.637 **
Current Mental Health Services	5 (15.2%)	1.886	1.245	0.001	0.157		0.262	0.001
Mental Health Services (Past)	13 (39.4%)	0.016	1.172	4.406 *	1.528		5.629 **	4.406 *
*Current Self-Reported Medication Use*								
Ssri’s	7 (21.2%)	0.090	0.287	0.287	0.007		0.012	0.044
Aspirin	11 (33.3%)	1.320	1.450	0.090	0.687		0.071	0.253
Statins	9 (27.3%)	0.557	0.556	0.007	0.229		0.053	0.132
High Blood Pressure Medication	16 (48.5%)	0.831	0.267	1.410	1.637		1.962	0.046
Contraceptives	1 (3%)	3.223	0.277	0.277	2.750		0.448	1.586
Hormone Replacement	5 (15.2%)	0.797	1.244	5.305 *	0.157		2.460	1.244
Sleep Hours Per Day (≥8 or <6)	7 (21.2%)26 (78.8%)	1.676	2.392	1.172	0.755		3.030	1.172
Smoking Behavior in the Past 6 Months (Yes)	4 (12.1%)	0.001	7.880 **	2.25	1.70		0.836	0.214
Alcohol Consumption in the Past 6 Months (Yes)	10 (30.3%)	5.18 *	0.663	3.03	0.054		2.63	5.62 *

* *p* < 0.05; ** *p* < 0.01.

**Table 2:** Correlations among immune infiltration markers and known behavioral and biological mediators/moderators.

Variables	CD68 Macrophages	CD3 T-Cells	CD19 B-Cells
***Known biological mediators/moderators***			
Age	0.090	0.528 **	−0.460 *
BMI	0.184	0.2816	−0.012
Menopause status	9.000 *	21.500	21.000
Tumor size	0.511	−0.118	0.011
Cancer treatment: Chemotherapy	0.000 **	9.000	4.000
Cancer treatment: Radiation Therapy	13.000	12.000	12.000
Cancer treatment: Hormonal Therapy	10.000	10.000	9.000
Lymph node status	3.000 *	19.000	7.000
SSRI’s	41.000	167.000	40.000
Aspirin	15.000	51.5000	41.000
Statins	40.000	64.000	45.000
High blood pressure medication	33.000	44.000	39.000
Contraceptives	2.000	4.000	2.000
Hormone replacement	26.000	23.000	24.000
**Sleep hours per day**	33.000	28.500	35.000
***Known behavioral mediators/moderators***			
Current use of stress management skills	37.000	42.5000	39.000
Mental health services (present)	16.000	9.500	1.000 *
Mental health services (past)	15.000 *	35.500	39.000
Smoking behavior	22.000	21.000	25.000
Alcohol consumption	29.000	39.000	37.000

* *p* < 0.05; ** *p* < 0.01.

**Table 3:** Correlations among depression, anxiety, exposure to SEA, and immune cell infiltrates.

Variables	CD68 Macrophages	CD3 T-Cells	CD19 B-Cells
PHQ-8 total score (depression symptoms)	0.224	0.466 *	0.581
Depression symptoms (current)	20.000	33.500	33.000
Hx. of Depression	32.000	49.000	44.000
GAD-7 total score (anxiety symptoms)	0.098	0.013	0.189
Adverse Childhood Events (ACE, total score)	0.805	0.886	0.457
Trauma History Questionnaire (THQ, total score)	−0.054	0.015	0.173
THQ/Crime-related events (total score)	0.163	0.067	0.216
THQ/Crime-related events (average age)			
THQ/General disasters and trauma (total score)	-	0.166	0.109
THQ/General disasters and trauma (average age)			
THQ/Physical and Sexual experiences (total score)	0.067	0.288	−0.034
THQ/Physical and Sexual experiences (average age)			
Comorbid ACE + Hx. of Depression	30.000	32.500	26.000
Comorbid ACE + Anxiety	20.000	23.500	16.000
Comorbid THQ/Crime + Hx. of Depression	24.000	28.000	34.000
Comorbid THQ/Crime + Anxiety	18.000	22.000	10.000 *
Comorbid THQ/Disaster and trauma + Hx. of Depression	32.000	49.500	34.000
Comorbid THQ/Disaster and trauma + Anxiety	20.000	23.500	16.000
Comorbid THQ/Physical and sexual abuse + Hx. of Depression	32.000	49.500	34.000
Comorbid THQ/Physical and sexual abuse + Anxiety	12.000	8.000 *	20.000

* *p* < 0.05.

## Data Availability

The data presented in this study will be available in this article.
